# Reliability and agreement during the Rapid Entire Body Assessment: Comparing rater expertise and artificial intelligence

**DOI:** 10.1371/journal.pone.0323262

**Published:** 2025-05-09

**Authors:** Denise Balogh, Xiaoxiao Cui, Monique Mayer, Niels Koehncke, Ryan Dueck, Angelica E. Lang

**Affiliations:** 1 Canadian Center for Rural and Agricultural Health, University of Saskatchewan, Saskatoon, Saskatchewan, Canada; 2 College of Veterinary Medicine, South China Agricultural University, Guangzhou, Guangdong, China; 3 Small Animal Clinical Sciences, Western College of Veterinary Medicine, University of Saskatchewan, Saskatoon, Saskatchewan, Canada; 4 Department of Medicine, College of Medicine, University of Saskatchewan, Saskatoon, Saskatchewan, Canada; 5 IPM Occupational Therapy, Saskatoon, Saskatchewan, Canada; Arak University of Medical Sciences, IRAN, ISLAMIC REPUBLIC OF

## Abstract

The purpose of this study was to examine the reliability and agreement between human raters (novice, intermediate, and expert) and TuMeke Risk Suite when assessing work with the Rapid Entire Body Assessment (REBA). Twenty-one videos portraying veterinarians performing an equine radiograph were assessed with REBA by human raters and TuMeke Risk Suite (ergonomic artificial intelligence software). Intra-rater reliability of the final REBA score was highest for TuMeke Risk Suite (ICC = 1.0), then the expert rater (ICC = 0.89 (0.78–0.95)), and lowest for the novice rater (ICC = 0.51 (0.25–0.74)). Agreement between the expert rater and TuMeke Risk Suite was highest for scores of the trunk, leg, and upper arm, and lowest for the neck, wrist, and lower arm. The REBA tool in TuMeke Risk Suite may be of benefit to less experienced users to enhance reliability of their REBA assessments, especially when the trunk, legs, and upper arm are of primary interest.

## Introduction

Musculoskeletal disorders are prevalent across numerous occupations, with risk factors for injury including non-neutral body postures, repetition, and high force. The need to assess occupational risk has led to the development of various observational ergonomic assessment tools, such as the Rapid Upper Limb Assessment (RULA), NIOSH lifting equations, Posture, Activity, Tools and Handling (PATH), Revised Strain Index (RSI), and others [1–5]. In particular, the Rapid Entire Body Assessment (REBA) is a relatively simple postural analysis tool for assessing work activities that require full body contributions and their associated level of musculoskeletal risk [6]. The REBA scoring sheet provides scores for each body region assessed (neck, trunk, legs, upper arm, lower arm, and wrist), an overall score, as well as a final level of risk/action level. REBA is designed to be sensitive to the musculoskeletal risks in various tasks and jobs, as the required force/load, stability during the task, the static or dynamic nature of the task and coupling during manual materials handling are all considered in the final score [6].

REBA is a popular tool used extensively over the years to assess postural risk factors during work [7,8]. REBA is used in professional ergonomics assessments, and it has also been widely employed in academic research for the quantification of risk factors in various occupations [7,9,10]. For example, REBA has been used to quantify risk in manual dairy farm workers [10], chair-side dental students [9], rick-shaw operators [11], and surgical residents [12] among many others [7].

Traditionally, observational tools such as REBA are conducted by visual assessment, either in person or of video recordings. However, the recent rise of various artificial intelligence (AI) software that automatically compute scores for observational ergonomic risk assessments based on a video input has provided an alternative [8,13–16]. An example of one such software is TuMeke Risk Suite; TuMeke Risk Suite is a proprietary, commercially available AI ergonomic assessment software developed by TuMeke Ergonomics Inc. [17]. TuMeke Risk Suite requires video input; the posture of the subject within the video is then tracked with a deep learning algorithm, providing estimates of joint angles. The software is capable of providing automatic REBA, RULA, RSI or NIOSH Lifting Equation scores, a visual overlay of the subject within the video which is color-coded to indicate level of risk by body part, joint angles presented in chart format, and suggestions for decreasing the overall level of risk [17]. TuMeke Risk Suite allows for fast, large scale ergonomic assessments and estimates of injury risk without interference with work, and without the need for additional technology such as wearables. However, despite these benefits and the current use of TuMeke Risk Suite by professional ergonomists, no assessment of the TuMeke Risk Suite reliability or validity can be found in the literature.

In Canada, practicing ergonomists will have a standard of education and professional experience to be considered experts [18]. However, the same depth of practical ergonomics experience may not always be present in researchers interested in employing ergonomic tools to study occupational risk factors for injury, despite potentially high theoretical knowledge. Various studies have suggested lower performance in novice users of ergonomic assessment methods [19–22]. For example, when asked to judge how likely the subject of a written scenario was to develop an upper limb disorder, practicing ergonomists demonstrated superior performance in comparison to university students belonging to an ergonomics class [21]. Stanton and Young [20] tested the reliability and validity of 10 different ergonomic assessment methods (e.g., checklists, observation, layout analyses, etc.) when conducted by novices (university students). Lower intra and inter-rater reliability, especially for the more complex ergonomic assessment methods, led authors to suggest that caution should be heeded when ergonomics assessment methods are used by a novice [20]. Employing an AI ergonomic assessment software to automatically compute assessment scores from tools such as REBA may provide a more reliable alternative for the novice ergonomics assessor when conducting ergonomic evaluations. This may be particularly relevant to research where large-scale studies are often needed, but expertise with observational methods may vary. AI software, if reliable, can potentially lead to higher quality ergonomics research. The outputs of some AI ergonomic assessment tools have been compared against expert human ratings to examine the validity of the tools [13,14,16]. Good agreement between the RULA scores generated from an AI video-based ergonomics software and expert human reviewers was reported [14]. However, given the potential range of expertise by users of ergonomic assessment tools, it is also important to examine the agreement between AI and humans of different expertise levels, to better inform the use of the AI assessment tool.

The purpose of this study was to assess the reliability of REBA when used by raters of varying expertise, and to test the agreement between the REBA scores generated by the human raters with the REBA scores calculated by TuMeke Risk Suite. We hypothesized that reliability would increase with expertise for the human raters and agreement with TuMeke Risk Suite would be highest for the expert rater.

## Methods

### Participants

This study was approved by the University of Saskatchewan Research Ethics Board (Beh ID 4155); informed consent was obtained by all participants prior to their involvement. This study was a secondary analysis of a larger study of ergonomic risk factors of veterinary medical workers taking radiographs of horses. Participants were recruited from a convenience sample from the University of Saskatchewan Western College of Veterinary Medicine and the University of Regina. Inclusion criteria for participation included being over 18 years of age, currently a veterinary medical worker, and involved in acquiring radiographs of horses. The data from 3 female veterinary medical workers (1 senior clinician, 1 junior clinician, and 1 senior veterinary student; mean (SD) age = 29 (7.8) years, weight = 67 (8.7) kg, height = 169.3 (15) cm) from the larger study were used for the analysis of reliability and agreement in the present study.

### Data collection

The veterinarians were filmed with a single camera (Honor 20 Lite, Huawei, Shenzhen, China) while taking equine radiographs as part of their natural workday. In total, 21 videos of the three veterinarians were collected to establish the reliability and agreement of REBA outputs calculated by TuMeke Risk Suite (TuMeke ergonomics, San Mateo, CA) and human raters in the present study. The 21 videos each portrayed a veterinarian imaging an anatomical location on the horse’s lower extremity (hoof, pastern, fetlock, metatarsal, hock or knee), which required the veterinarian to assume different body positionings when radiographing at each of these locations. Each video clip (mean 24.6 ± 13.8 sec) captured the veterinarian moving into position, completing the radiograph scan, and then moving out of position upon completion of the radiograph. All videos were obtained by the same researcher for consistency.

### Data analysis

**Fig 1:**
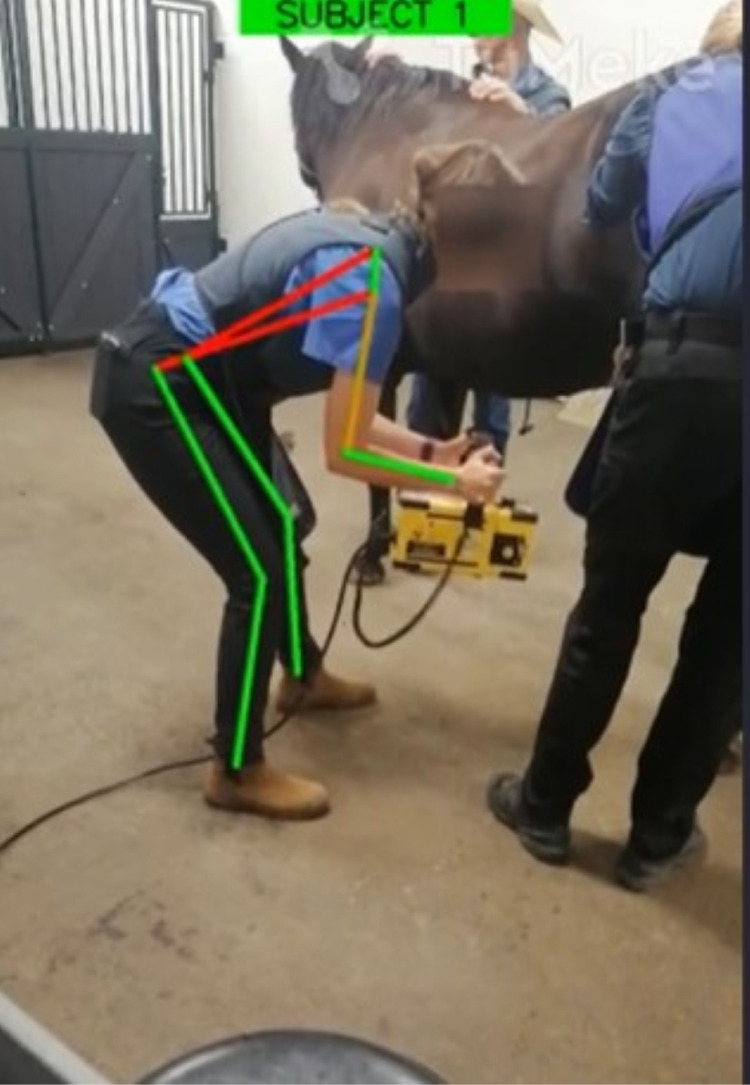
Example of TuMeke overlay in video frame selected for REBA analysis.

The 21 videos were inputted into TuMeke Risk Suite and the final REBA score (possible range: 1–15), level of risk (five categories), and score for each anatomical location (neck, trunk, legs, upper arm, lower arm, and wrist) as indicated by the REBA Employee Assessment Worksheet were calculated (Fig 1). For each video rating within TuMeke Risk Suite, other relevant inputs were manually selected by the researcher responsible for capturing the videos, including “Add Force/Load Score”, “Add Coupling Score” and “Activity Score”. The “Add Force/Load Score” was set to + 1 for each video due to the 15–16 lbs weight of the handheld radiograph ray machine. The “Add Coupling Score” was set to + 0 as the radiograph machine was equipped with a handle that allowed for good hand positioning and grip power, and the “Activity Score” was set to + 1 for each video due to the rapid large range changes in posture that occurred as the veterinarian moved into and out of position to complete the x-ray. Additionally, the specific timepoint within each video that was selected by TuMeke for REBA analysis was documented. The selected timepoint corresponds with the frame determined to have the highest level of risk. As TuMeke Risk Suite is a proprietary software, the deep learning algorithms used by the software are not available to the authors to describe in this manuscript. However, the formulas used to calculate the joint angles can be found in the TuMeke Risk Suite white paper [15], and this may be of interest to readers to further their understanding of the software.

Three raters with varying levels of expertise (novice, intermediate, and expert) in ergonomic analysis also assessed the videos to establish reliability and agreement between TuMeke Risk Suite and human REBA analysis. The raters included one PhD student with 2 years of experience in biomechanics and ergonomics research (novice expertise), one assistant professor with 10 years of experience in biomechanics and ergonomics research and teaching (intermediate expertise), and one practicing ergonomist (expert expertise) with over 15 years of practical experience. University ergonomics students and practicing ergonomists have previously been categorized into ‘novice’ and ‘expert’ expertise levels in research relating to expertise level and ergonomic assessments [20,21]. Each rater visually assessed the 21 videos with the REBA scoresheet three separate times (at least one week apart). The REBA analysis was conducted at the timepoint determined to represent the highest level of risk within each video clip, chosen separately by each rater during each of the 3 repeated assessments; this selected timepoint was documented. The raters were blinded to each others’ scores, as well as to the scores calculated by the TuMeke Risk Suite. The order of videos was randomized from session to session, as well as between each rater within each session to eliminate any potential learning bias.

Additionally, to control for differences in scores due to different timepoint selections, the three human raters also scored still images extracted from each of the videos at the exact timepoint selected by TuMeke Risk Suite during a fourth round of ratings. An example of the REBA score sheets used by TuMeke Risk Suite and by the human raters can be found in S3 Appendix.

### Statistical analysis

Intra-rater reliability of final REBA score, final level of risk and score for each anatomical location was assessed with ICCs and 95% confidence intervals for each rater (novice, intermediate, expert, and TuMeke) across the three video rating sessions based on a single rater, absolute-agreement, 2-way mixed-effects model. Similarly, inter-rater reliability of final REBA score, final level of risk and score for each anatomical location was determined from ICC estimates and 95% confidence intervals between the three raters based on a single rater, absolute agreement, 2-way random-effects model [23]. Interpretation of the ICCs was based on the following criteria: > 0.5 = poor reliability; 0.5 to 0.75 = moderate reliability; 0.75 to 0.9 = good reliability; > 0.9 = excellent reliability [23].

Agreement between the TuMeke Risk Suite and each human rater for all REBA outcomes (final REBA score, final level of risk, and score for each anatomical location) was calculated for the videos scored at self selected timepoints, as well as for the still images extracted from each video at the timepoint selected by TuMeke Risk Suite. For the final REBA score, final level of risk, as well as the scores for neck, trunk, legs, upper arm and wrist, agreement was calculated using a linear weighted kappa. The Cohen’s kappa was used to determine agreement involving the lower arm due to the presence of only two categories (alpha = 0.05; p-values greater than 0.05 signify no agreement) [24]. The kappa statistic was interpreted according to the following criteria: < 0 = poor agreement; 0 to 0.20 = slight agreement; 0.21 to 0.40 = fair agreement; 0.41 to 0.60 = moderate agreement; 0.61 to 0.80 = substantial agreement; 0.81 to 1 = almost perfect agreement [25]. Agreement between TuMeke Risk Suite and each human rater for the final REBA score was also determined through Bland-Altman plots. The difference in the Bland-Altman plots were calculated as the TuMeke REBA score minus human REBA score. All analyses were performed using SPSS (v28, SPSS Inc, Chicago, IL).

## Results

REBA ratings for all videos by all raters were within the range of 5–10 (Table 1), indicating all tasks assessed were at medium (Level 3) to high risk (Level 4).

**Table 1:** Final REBA score (mean (SD)) averaged across the three video rating sessions for each video for both human and TuMeke Risk Suite raters.

	Final REBA Score			
Video Number	Novice	Intermediate	Expert	TuMeke Risk Suite
1	6.3 (1.2)	7.3 (0.6)	7 (0)	7 (0)
2	7 (0)	7.7 (0.6)	7.7 (0.6)	7 (0)
3	5.7 (1.2)	6.3 (1.2)	5 (0)	9 (0)
4	7 (0)	5 (0)	7 (0)	7 (0)
5	7 (0)	8.3 (0.6)	7 (0)	8 (0)
6	5 (0)	5.3 (0.6)	5 (0)	5 (0)
7	6.3 (1.2)	6.7 (1.5)	5 (0)	7 (0)
8	5.7 (1.2)	6.7 (1.5)	6.3 (1.2)	8 (0)
9	7.3 (0.6)	8 (0)	7 (0)	10 (0)
10	7.3 (0.6)	9 (0)	5 (0)	5 (0)
11	7.7 (0.6)	8.3 (1.2)	7.3 (0.6)	8 (0)
12	6.7 (1.2)	6 (0)	7 (0)	8 (0)
13	7 (0)	6.7 (0.6)	9 (0)	8 (0)
14	8 (1.7)	9.7 (0.6)	7 (0)	10 (0)
15	7.7 (0.6)	8 (0)	8 (0)	8 (0)
16	7 (0)	9 (0)	8 (0)	9 (0)
17	8 (0)	6 (1)	7.7 (0.6)	9 (0)
18	6.3 (1.2)	6.3 (0.6)	7 (0)	9 (0)
19	7.3 (0.6)	7.3 (2.1)	8 (0)	10 (0)
20	7.3 (0.6)	6 (1)	7.3 (1.2)	10 (0)
21	9.3 (0.6)	9 (0)	9 (0)	10 (0)

### Intra-rater reliability

The intra-rater reliability of all raters (human and TuMeke Risk Suite) is presented in Table 2. Out of the human raters, the expert rater demonstrated the highest reliability for the final REBA score and level of risk (ICCs = 0.89 and 0.73, respectively), while the novice rater was the least reliable (ICCs = 0.51 and 0.38, respectively). The TuMeke Risk Suite provided perfect reliability (ICCs = 1.00) for all REBA scoring categories, in that identical scores were calculated when the same videos were fed into the software multiple times.

**Table 2:** Intra-rater reliability of human raters and TuMeke Risk Suite across the three video rating sessions at self selected timepoints.

	Novice Rater	Intermediate Rater	Expert Rater	TuMeke Risk Suite
	ICC (95% CI)	ICC (95% CI)	ICC (95% CI)	ICC (95% CI)
Final REBA Score	0.51 (0.25-0.74)	0.66 (0.43-0.83)	0.89 (0.78-0.95)	1.00
Final Level of Risk	0.38 (0.12-0.64)	0.63 (0.40-0.81)	0.73 (0.53-0.87)	1.00
Neck	0.48 (0.23-0.72)	0.63 (0.40-0.81)	0.76 (0.57-0.88)	1.00
Trunk	0.54 (0.29-0.76)	0.76 (0.57-0.88)	0.61 (0.37-0.80)	1.00
Leg	0.80 (0.63-0.90)	0.56 (0.30-0.77)	0.96 (0.91-0.98)	1.00
Upper Arm	0.85 (0.73-0.93)	0.69 (0.48-0.85)	0.66 (0.44-0.83)	1.00
Lower Arm	0.59 (0.34-0.79)	0.51 (0.25-0.73)	0.93 (0.86-0.97)	1.00
Wrist	1.00	0.68 (0.46-0.84)	1.00	1.00

### Inter-rater reliability

Inter-rater reliability of human raters when scoring the videos at self selected time points is presented in Table 3. ICCs indicated that the inter-rater reliability for final REBA score and final level of risk was poor (ICCs = 0.36 and 0.22, respectively).

**Table 3:** Inter-rater reliability of the novice, intermediate and expert human raters for REBA ratings at self selected timepoints of perceived highest risk in the videos.

	ICC (95% CI)
Final REBA Score	0.36 (0.21-0.52)
Final Level of Risk	0.22 (0.07-0.38)
Neck	0.36 (0.20-0.51)
Trunk	0.47 (0.32-0.61)
Leg	0.70 (0.59-0.80)
Upper Arm	0.41 (0.25-0.56)
Lower Arm	0.40 (0.24-0.55)
Wrist	0.74 (0.64-0.83)

### Agreement

The agreement between human raters of varying expertise and TuMeke risk suite during scoring at self selected timepoints (data in S1 Dataset) is presented in Table 4. The kappa statistic revealed agreement ranging from poor to moderate for the novice and expert, and poor to fair for the intermediate rater across outcomes. The expert rater exhibited higher agreement for final REBA score, final level of risk, as well as for the leg, upper arm, lower arm, and wrist. The highest agreement with TuMeke Risk Suite for all three human raters occurred during ratings of the leg.

**Table 4:** Agreement of REBA scores between human raters and TuMeke Risk Suite at self selected timepoints within the videos.

	Novice	Intermediate	Expert
	Kappa (p-value)	Kappa (p-value)	Kappa (p-value)
Final REBA Score	0.20 (<0.01)	0.15 (0.03)	0.25 (<0.01)
Final Level of Risk	0.21 (0.02)	0.10 (0.38)	0.24 (0.01)
Neck	-0.02 (0.85)	-0.44 (<0.01)	-0.27 (0.01)
Trunk	0.39 (<0.01)	0.26 (<0.01)	0.24 (<0.01)
Leg	0.47 (<0.01)	0.36 (<0.01)	0.49 (<0.01)
Upper Arm	0.19 (0.01)	0.19 (0.01)	0.26 (<0.01)
Lower Arm	-0.12 (0.17)	-0.02 (0.84)	0.06 (0.60)
Wrist	-0.12 (0.30)	-0.02 (0.84)	0.02 (0.86)

The agreement of REBA scores when the human raters scored still images extracted at the timepoints selected by TuMeke Risk Suite (data in S2 Dataset) are presented in Table 5. For the expert rater, agreement with TuMeke improved for each REBA outcome over the agreement calculated from ratings at self selected timepoints, with agreement ranging from poor to substantial. The expert rater had the highest levels of agreement for final REBA score; the novice rater showed the highest agreement with TuMeke Risk Suite for final level of risk. The highest agreement between TuMeke Risk Suite and all three human raters was for the trunk, which was substantial agreement.

**Table 5:** Agreement of ratings from still images between human raters and TuMeke Risk Suite at timepoints selected by TuMeke Risk Suite.

	Novice	Intermediate	Expert
	Kappa (p-value)	Kappa (p-value)	Kappa (p-value)
Final REBA Score	0.20 (0.12)	0.08 (0.36)	0.35 (<0.01)
Final Level of Risk	0.49 (0.02)	0.12 (0.45)	0.28 (0.13)
Neck	0.02 (0.88)	-0.12 (0.53)	0.18 (0.38)
Trunk	0.68 (<0.01)	0.68 (<0.01)	0.63 (<0.01)
Leg	0.63 (<0.01)	0.65 (<0.01)	0.57 (<0.01)
Upper Arm	0.14 (0.20)	0.16 (0.11)	0.53 (<0.01)
Lower Arm	0.23 (0.28)	0.13 (0.54)	0.23 (0.28)
Wrist	0.15 (0.19)	-0.18 (0.24)	-0.09 (0.42)

**Fig 2:**
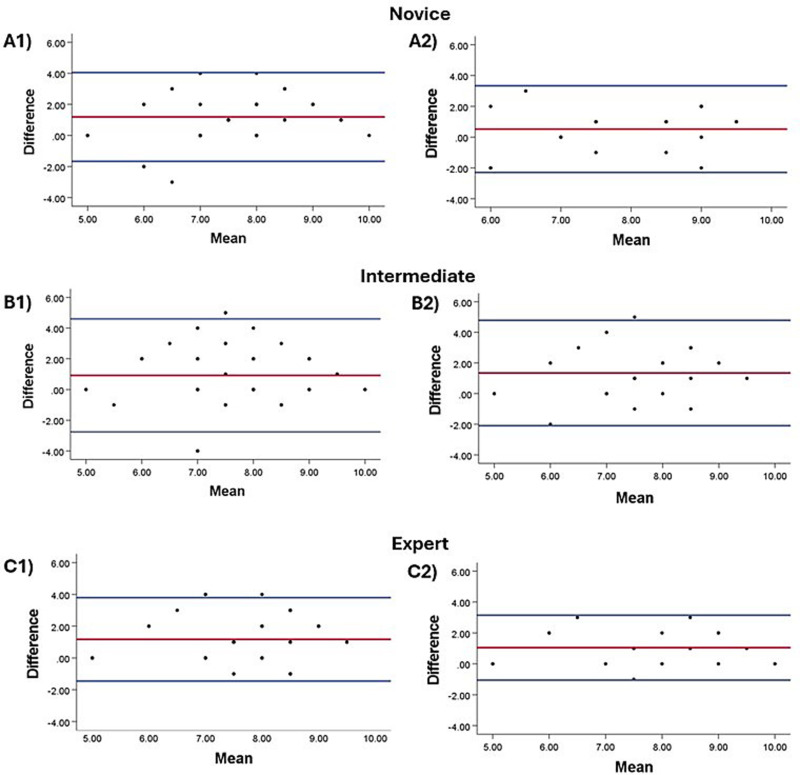
Bland-Altman plots (mean = red line; limits of agreement = blue lines) displaying agreement between each human rater (novice, intermediate, expert) and TuMeke for the Final REBA Score. The agreement between each human rater and TuMeke during scoring at self-selected timepoints are presented in A1, B1), and C1). The agreement at timepoints selected by TuMeke are presented in A2), B2), and C2). The different numbers of points from plot to plot are due to overlapping datapoints.

Bland-Altman plots depicting the level of agreement between the human raters and TuMeke for the final REBA score mirrored the agreement determined with the Kappa statistic (Fig 2). Specifically for the expert rater, the limits of agreement were wider when agreement was calculated at self-selected timepoints (Fig 2 C1) but were narrower when agreement was calculated at the timepoints selected by TuMeke (Fig 2 C2). This suggests improved agreement when controlling for variability in the rated posture.

## Discussion

In this study, intra and inter-rater reliability of raters with varying expertise as well as agreement between human raters and TuMeke Risk Suite was examined. While several other studies have previously tested reliability of REBA, this was the first study to examine reliability and agreement of REBA based on expertise and compared to ergonomic AI software.

### Reliability

As hypothesized, intra-rater reliability for final REBA score and final level of risk was highest for the expert rater and lowest for the novice rater, suggesting that with increased expertise there is increased consistency in scoring REBA. This is unsurprising, as lower performance and reliability have been documented in the novice ergonomic tool user [19–22]. The decreased consistency of the novice rater may be due to several reasons. These include lack of practical experience and application of the REBA assessment tool, less familiarity with high risk postures, and possibly a decreased ability to identify the pertinent information from a video showing a complex dynamic movement [20]. In contrast, the expert rater had extensive practical experience with using the REBA tool to conduct ergonomic assessments involving a wide range of working postures. Consequently, there is room for improvement in the reliability of scoring REBA for primarily the novice and intermediate raters. The intra-rater reliability of TuMeke Risk Suite was perfect for all categories, demonstrating its advantage in reliability when compared to the human raters, especially novice raters.

Intra-rater reliability findings are likely influenced by expertise and study design. For example, intra-rater reliability of REBA ratings of 7 novice raters and 1 expert rater, pooled together for analysis, reported ICC values of 0.93 for the final REBA score [26]. An ICC score of 0.93 is markedly higher than the intra-rater reliability found in this study, even for the expert rater. However, the previous work had the raters complete their repeated assessment immediately one after another [26]; in the present study, ratings occurred at least one week apart to minimize any learning effects. Using a similar approach, the intra-rater reliability of examiners, described as researchers with 5–7 years experience in occupational health research, was also examined in the Brazilian-Portuguese version of REBA using repeated scoring one week apart [27]. Intra-rater reliability for final REBA score and final level of risk was found to be moderate and poor, respectively, which aligns more closely with the reliability of the novice and intermediate raters in the present study. As evidenced by [27] and the results of the present study, timing of the repeated assessments may influence results, and lower reliability of REBA assessments may be found in the health researcher with potentially limited practical experience with REBA in comparison to the practicing ergonomist. The intra-rater reliability of TuMeke Risk Suite was perfect across all categories, suggesting that the software may be used for highly consistent scoring of REBA in individuals who may have high theoretical knowledge of ergonomics, but less practical experience using the tool.

In terms of inter-rater reliability of the human raters, reliability for final REBA score and final level of risk was poor. This lower inter-rater reliability is not unexpected, considering the range of expertise level among the raters. This finding suggests that caution should be heeded when interpreting REBA scores from multiple raters, especially in the case of raters with varying expertise levels. Ratings from a group of examiners at the same expertise level, even of novice examiners, may increase inter-rater reliability [26]. However, both inter-rater reliability of REBA scored by novice raters (university students from an ergonomics class) [28] and agreement of health researchers with 5–7 years experience [27] has been reported as only slight to moderate. Therefore, despite being marginally higher among groups of raters with similar expertise level, inter-rater reliability of REBA scores is frequently low across human raters.

### Agreement

Agreement between human raters of varying expertise and TuMeke Risk Suite was calculated for the scores obtained during REBA ratings of the video clips at the timepoints selected by each rater, which were perceived to be the highest risk frames in the videos. While rating the video clips at self-selected time points may be more reflective of a natural REBA assessment, it was necessary to conduct the ratings at TuMeke selected timepoints to control for variability of the rated posture. As such, agreement was also calculated for the REBA ratings of still images extracted from each video at the timepoints selected by TuMeke Risk Suite. Unsurprisingly, the level of agreement between the expert rater and TuMeke Risk Suite increased for all outcomes when conducting the REBA assessment for still images at TuMeke selected timepoints. The impact of reduced variability was less definitive for the novice and intermediate raters, as agreement with TuMeke increased in some outcomes but decreased in others when scoring the still images at TuMeke selected timepoints.

Reliability and agreement also varied by body region. The highest agreement with TuMeke occurred during scoring of the trunk (substantial agreement). A previous study also reported substantial agreement between expert raters and an ergonomic AI tool for the RULA trunk score [16]. It is possible that the typically slower and more exaggerated movement of the trunk is easier to detect by both humans and AI tools. However, despite the highest inter-rater reliability between the three human raters occurring for the wrist, the agreement of the wrist score between humans and TuMeke was very low. Observational, video-based assessment of the wrist has previously been reported to be substantially influenced by the view due to artifacts such as image parallax [29]. The videos and static images analyzed by all raters (human and AI) in the present study were all side or oblique views, suggesting difficulty for raters to reliably assess wrist posture. As indicated by the levels of agreement between the expert rater and TuMeke, the use of TuMeke may be more appropriate when the trunk, legs, or upper arm are of primary interest to the researchers or ergonomist; TuMeke scores for the wrist, lower arm and neck may require human verification. Further validation is needed to confirm the accuracy of TuMeke Risk Suite.

The use of the REBA tool in TuMeke Risk suite may be beneficial to the novice user when conducting ergonomic assessments, specifically when monitoring select body regions. This finding is especially important for the application of the REBA assessment in ergonomics research. Numerous research studies use REBA to investigate ergonomic risk, but these investigations are often conducted by university students or researchers (novices) who may lack practical experience with ergonomic assessments [7,9–11]. Using a tool such as TuMeke may help to ensure consistency in ratings amongst novices, leading to more accurate results, and more robust strategies to improve worker safety.

### Limitations

This study is not without limitations. First, only three raters, each with a different level of expertise, contributed to the ratings in this study, potentially reducing the generalizability of our findings. However, identical approaches regarding the inclusion of single raters of varying levels of expertise are accepted in previous reliability research in the ergonomics and biomechanics field [30,31]. Also, the intra-rater reliability in the present study closely reflects the intra-rater reliability of REBA scores documented in other studies of similar structure, providing confidence in our results [27]. While inter-rater reliability was tested between the three raters of different expertise levels, inter-rater reliability within each expertise level was not examined. However, it is expected that the inter-rater reliability within a larger sample of raters of similar expertise would be marginally higher than the reliability between raters of varying levels of expertise [27,28]. Additionally, while agreement and reliability between human raters and TuMeke Risk Suite were tested in this study, validity was not. Consequently, it can not be said with certainty that TuMeke Risk Suite provides more accurate REBA ratings over the human raters. However, as TuMeke Risk Suite is currently being used by researchers and industry to conduct ergonomics assessments, it is imperative that high reliability of this A.I. software in comparison to human REBA ratings is established. As TuMeke Risk Suite also outputs joint angles alongside REBA scores, future research will validate TuMeke Risk Suite against an objective motion capture system to determine accuracy of this software. Finally, this study focused on the reliability and agreement of the REBA tool only; the other tools available within TuMeke Risk Suite, including RULA, RSI, and NIOSH lifting equations, were not assessed. Consequently, the results of this study are specific to the REBA tool and are not applicable to the other assessment tools available within TuMeke Risk Suite. Examining the reliability and accuracy of a wider battery of AI-led ergonomic assessment tools is an area for future research.

## Conclusion

The reliability and agreement between human raters of varying expertise level and TuMeke Risk Suite were investigated. The highest intra-rater reliability of REBA scores occurred for the expert rater, with lower reliability in the novice and intermediate raters. The REBA tool in TuMeke Risk Suite may be useful for individuals with less practical experience with conducting ergonomic assessments in increasing the reliability of their assessments. Additionally, the REBA tool in TuMeke Risk Suite may be used with most confidence when the trunk, legs, and upper arm are of greatest interest and importance to the user. Further validation of TuMeke Risk Suite is needed to confirm its accuracy.

## Supporting information

S1 Dataset Dataset corresponding to REBA scores of videos at self-selected timepoints. (XLSX)

S2 Dataset Dataset corresponding to REBA scores of still images at timepoints selected by TuMeke Risk Suite. (XLSX)

S3 Appendix Example REBA scoresheets used by TuMeke and the human raters. (DOCX)
