# Supplementary material for: Reliability and agreement during the Rapid Entire Body Assessment: Comparing rater expertise and artificial intelligence
Source: PLoS One. 2025 May 9;20(5):e0323262. doi: 10.1371/journal.pone.0323262 (PMC12063896; doi:10.1371/journal.pone.0323262)
Supplement: S3 Appendix — (DOCX) [file pone.0323262.s003.docx]

S3 Appendix. Example REBA scoresheets used by TuMeke and the human raters.


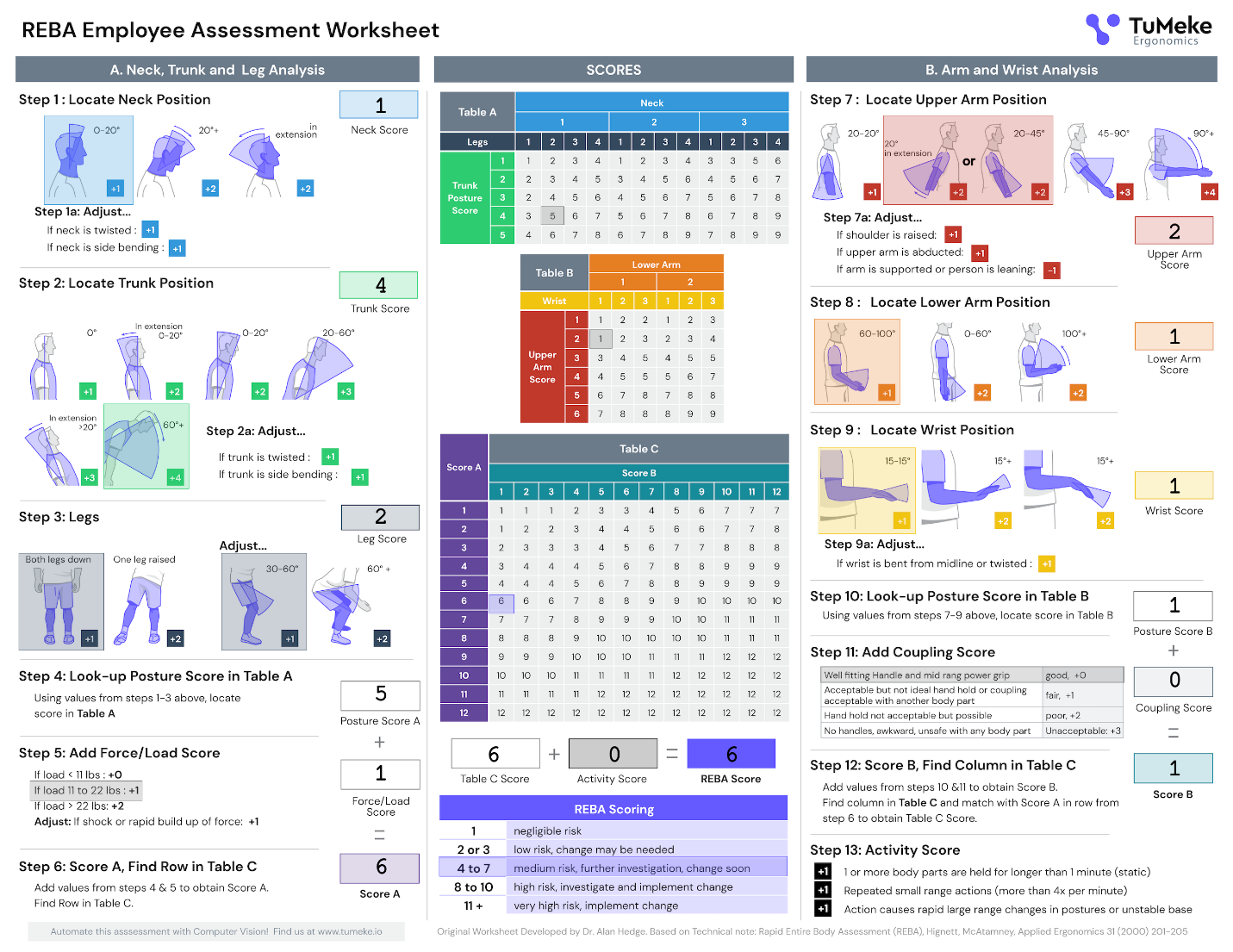


Example REBA assessment sheet automatically filled out by TuMeke.


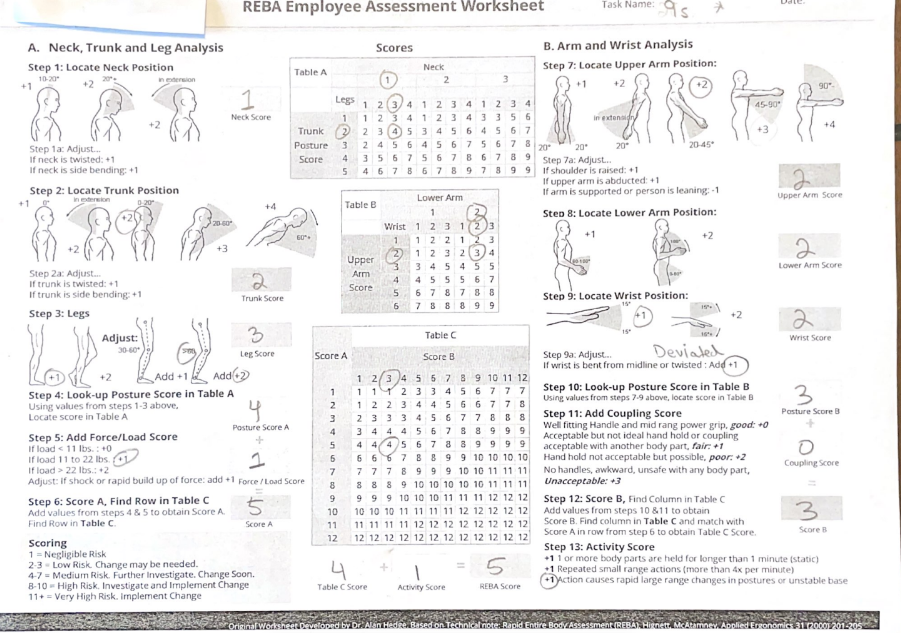


Example REBA assessment sheet filled out by a human rater.
